# Observed three dimensional distributions of enhanced turbulence near the Luzon Strait

**DOI:** 10.1038/s41598-021-94223-3

**Published:** 2021-07-21

**Authors:** Jianfeng Wang, Fei Yu, Feng Nan, Qiang Ren, Zifei Chen, Tongtong Zheng

**Affiliations:** 1 CAS Key Laboratory of Ocean Circulation and Waves, Institute of Oceanology, Chinese Academy of Sciences, Qingdao, 266071 China; 2 Center for Ocean Mega-Science, Chinese Academy of Sciences, Qingdao, 266071 China; 3 Pilot National Laboratory for Marine Science and Technology (Qingdao), Qingdao, 266237 China; 4 University of Chinese Academy of Sciences, Beijing, 100049 China

**Keywords:** Physical oceanography, Hydrology

## Abstract

Ocean turbulence can impact the transfer of heat, nutrients, momentum and sea level rise, which are crucially important to climate systems. The Luzon Strait, one of the mixing hotspots, is important for water exchange between the northeastern South China Sea and West Pacific. Here, for the first time, we carry out full-depth direct microstructure measurements surrounding the Luzon Strait to clarify the three-dimensional distributions of turbulence. We demonstrate that the turbulent kinetic energy dissipation rates in the upper and middle layers of the northeastern South China Sea are on the same order of magnitude as those in the West Pacific. The dissipation rates are only bottom enhanced near the rough topography of the South China Sea slope and Luzon Strait which is one order of magnitude larger than those at smooth area. The relevant bottom diapycnal diffusivity in the South China Sea is elevated in the West Pacific by a factor of three, instead of by two orders of magnitude as overestimated by indirect parameterization. These results may appear surprising in light of previous studies but are in fact consistent with predictions from internal wave-topography interaction theory.

## Introduction

The distribution and magnitude of ocean mixing impact the transfer of heat, nutrients, momentum, and sea level rise, which are crucially important to oceanic dynamics^1,2^. Recent work has shown that the distribution and magnitude of mixing may play critical roles in a variety of climate systems^3,4^. Open-ocean thermocline mixing is an order of magnitude smaller than the value required (10^–4^ m^2^s^−1^) to maintain global abyssal stratification^5^. Enhanced mixing with diffusivities higher than 10^–4^ m^2^s^−1^ has been observed over rough topographies^6–8^.

The Luzon Strait (LS) is the only deep channel connecting the northeastern South China Sea (NSCS) and the West Pacific (WP), with peak-to-peak baroclinic velocities and vertical displacements often exceeding 2 ms^−1^ and 300 m, respectively^9^. The topography surrounding the LS covers a wide range of bathymetric features, including two parallel ridges, deep basins, and steep continental slopes. Interacting with rough topography, energy converts from barotropic tides to internal tides^10^. Observations have revealed that the turbulent kinetic energy dissipation rate (*ε*) throughout the LS are some of the strongest ever measured^11^. Turbulent mixing in the LS drives water exchange between the NSCS and the WP, which is important for NSCS circulation and heat and salt budgets^12^.

Studies of temporal and spatial variations in turbulent mixing in the South China Sea (SCS) and LS have been carried out using microstructure observations and fine-scale parameterization. Limited by strong currents near the LS, previous microstructure measurements have mostly focused on the upper layer above 500 m^13–17^. Fine-scale parameterization has been used as a supplement for observations, and on this basis, it has been proposed that mixing in the NSCS is enhanced (10^–3^ m^2^s^-1^) by two orders of magnitude over that in the WP, with limited microstructure observation in the upper layer^18,19^. Elevated turbulent mixing (10^–2^ m^2^s^−1^) has been reported in the whole water column of the LS^19^. Some studies have suggested that enhanced mixing in the SCS and LS is mainly attributed to baroclinic tidal dissipation^11,20^.

Previous studies have greatly improved our knowledge of turbulent mixing in the SCS and LS. However, knowledge of mixing in deep layers of the SCS is mostly from fine-scale parameterization, which may introduce uncertainty in understanding turbulence variability in the deep ocean. Many existing ocean circulation models cannot explicitly resolve diapycnal mixing. Studies based on models have shown that a wide range of ocean dynamic processes are sensitive to the intensity and distribution of diapycnal mixing^21,22^. The results from numerical simulations based on simple parameterizations of diapycnal diffusivity should be treated with caution. Furthermore, numerical predictions of energy budgets cannot be tested without direct microstructure measurements from the LS. Confusion has persisted regarding the spatial distribution of turbulent mixing in the SCS and LS owing to the lack of in situ full-depth data because of the extremely challenging operating conditions^9^.

From our direct observation, direct results do not support the orders of magnitude of enhanced mixing in the SCS obtained from indirect measurements^18,19^. Our observations show that the dissipation rate in the NSCS is one order of magnitude higher than that in the WP and that the related diapycnal diffusivity in the NSCS is of the same order of magnitude as that in the WP. Enhanced dissipation is strongly related to the roughness and slope of topography.

## Results

### Field observations

**Figure 1:**
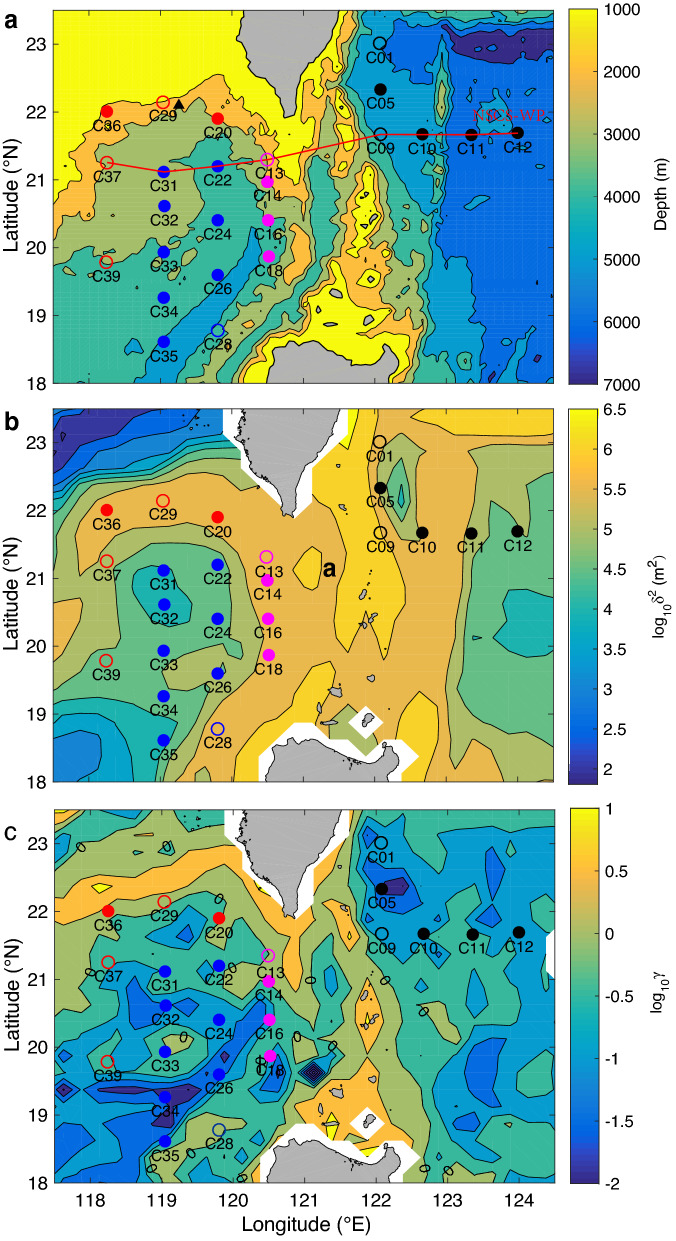
Map of the cruise area in the northeastern South China Sea (NSCS) and West Pacific (WP) showing microstructure measurement stations at the NSCS slope (red dots and circles), NSCS basin (blue dots and circles), and Luzon Strait (LS, magenta dots and circles) with the background topography (**a**), roughness (*δ*^2^, **b**) and slope criticality (*γ*) for semidiurnal tides (**c**). The red line (in panel a) indicates the cross-section in Fig. 2. Dots and circles represent microstructure profiles taken in 2018 and 2019, respectively. The black triangle near C29 is the station where the moored ADCP was deployed. Figures were plotted using MATLAB R2016b (http://www.mathworks.com/).

The objective of this paper is to clarify the full-depth three-dimensional distribution of small-scale turbulence based on direct measurements near the LS. Field observations were performed at 24 stations from May 29 to June 24, 2018, and from July 21 to August 5, 2019, in the NSCS and WP (Table 1), which covered a wide range of bathymetric and oceanographic conditions (Fig. 1). Measurements were taken using an expendable vertical microstructure profiler (VMP-X, Rockland Scientific Inc.), a conductivity-temperature-depth (CTD) profiler (911-plus, Sea-Bird Electronics), and two 300-kHz lowered acoustic Doppler current profilers (LADCPs) (Teledyne RD Instruments, one upward-looking and the other downward-looking). The VMP-X was equipped with 2 standard shear probes and 1 temperature sensor at the expendable ballast to fall at ~ 0.8 ms^−1^. Confined by rope length, traditional tethered microstructure profilers cannot reach deep depths under the influence of strong currents near the LS. The VMP-X works without a rope and releases the ballast when it reaches either a target depth (6000 m) or the seafloor^23^, which allows full-depth measurements at all stations. Without interference from the rope of the VMP-X, the CTD and LADCP were deployed together with the VMP-X, allowing quasi-synchronous sampling of CTD and microstructure data. Most CTD measurements can reach 50 m above the bottom, and the potential density (*σ*_θ_) was calculated based on CTD data.

**Table 1:** VMP-X stations and deployed time.

Station	Longitude (°E)	Latitude (°E)	Time to reach the bottom	Depth (m)
C36	118.2463	22.0033	05/29/2018 06:54:30	1550
C35	119.0567	18.6094	06/04/2018 13:22:33	4025
C34	119.0452	19.2598	06/05/2018 07:46:20	3661
C33	119.0483	19.9315	06/05/2018 15:16:39	3004
C32	119.0544	20.6133	06/05/2018 22:59:54	2767
C31	119.0509	21.1259	06/06/2018 07:15:02	2693
C20	119.8026	21.8971	06/06/2018 17:01:44	2199
C22	119.7927	21.197	06/07/2018 05:47:04	3390
C24	119.7984	20.3974	06/07/2018 14:23:01	3256
C26	119.8006	19.5973	06/08/2018 09:17:48	3895
C18	120.5195	19.8709	06/09/2018 20:36:31	3695
C16	120.5029	20.4092	06/10/2018 07:17:31	2550
C14	120.4976	20.9786	06/10/2018 17:16:29	1725
C10	122.662	21.6777	06/12/2018 19:49:53	4799
C12	123.995	21.6597	06/23/2018 14:53:34	5550
C11	123.3321	21.6613	06/23/2018 22:45:12	5450
C05	122.0889	22.3509	06/24/2018 11:26:22	4600
C29	119.0337	22.1479	12/10/2017 09:27:48	1082
C01	122.0733	23.0092	07/30/2019 07:27:24	4866
C09	122.0800	21.6693	08/03/2019 18:26:35	4702
C13	120.5228	21.3014	07/27/2019 13:49:37	1267
C20	119.8039	21.9545	07/27/2019 01:56:43	2223
C28	119.8047	18.7805	07/24/2019 08:16:53	3786
C29	119.0616	22.1692	07/21/2019 17:34:09	975
C37	118.2546	21.2557	08/04/2019 18:00:31	2115
C39	118.2439	19.788	08/05/2019 12:42:58	2664

A 75-kHz upward-looking moored acoustic Doppler current profiler (ADCP) was deployed at 22.10° N, 119.28° E, which is close to station C29, at a depth of 1135 m from July 25, 2017, to January 26, 2018. Velocity profiles were collected at an interval of 20 min using 8-m vertical bins. The first effective bin was located 16.7 m above the bottom. The ADCP measured velocity profiles within 500 m above the bottom. Gregg–Henyey–Polzin (GHP) scaling based on internal wave–wave interaction theory^25^ was applied to ADCP data to estimate the time series of the dissipation rate and to evaluate the variation in the dissipation rate. The buoyancy frequencies used in the GHP method are from global physical analysis and a coupled forecasting product^24^ modeled by the Met Office Coupled Atmosphere-Land–Ocean-Ice data assimilation system (CPLDA).

### Vertical distributions of turbulence along the NSCS-WP cross-section

**Figure 2:**
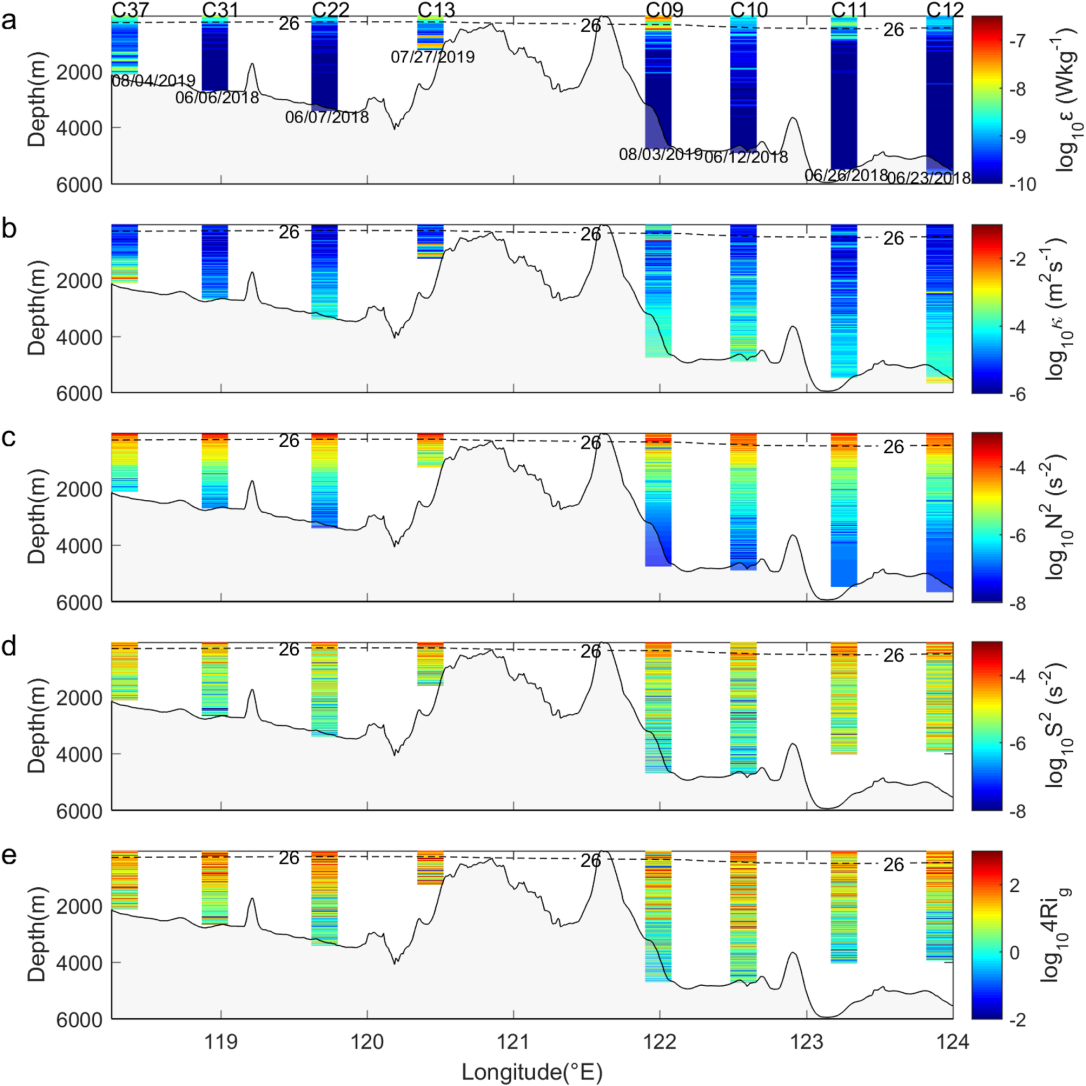
(**a**) Dissipation rate (*ε*), (**b**) diapycnal diffusivity (*κ*), (**c**) squared buoyancy frequency (*N*^2^), (**d**) shear squared (*S*^2^) and 4 times the gradient Richardson number (**e**) for the NSCS-WP cross-section in Fig. 1. The gray shading indicates bathymetry. In (**a**–**e**), the boundaries of the thermocline upper layer are taken as the 26-*σ*_θ_ isopycnal (dashed line), and the bin size is 10 m.

Cross-section along 21° N is widely used to compare turbulent mixing of the NSCS and WP^9,18^ since the internal tide energy flux is strongest within a beam that emanates from the LS between 20° N and 21° N. Cross-section NSCS-WP (red line in Fig. 1a), which is near cross-Sect. 21° N, was chosen to present the full-depth microstructure measurements (Fig. 2). Because depths in the NSCS and WP are different, by defining layers based on depth, one may bring unfair comparisons by comparing bottom-enhanced turbulence (500–1500 m in the NSCS) with mild turbulence in the middle layer (500–1500 m in the WP). Considering that turbulence is usually enhanced at the upper and bottom layers, three layers were defined, including the upper layer (50 m to the 26-*σ*_θ_ isopycnal^23^), middle layer (the 26-*σ*_θ_ isopycnal to 500 m above the bottom) and bottom layer (within 500 m above the bottom^3,19^).

Generally, the *ε* in the upper layer was higher than that in the other layers, with a maximum value of 1.32 × 10^–7^ Wkg^−1^ at C09 (Fig. 2a). Strong fine-scale velocity shear (Fig. 2d) was also observed in the upper layer. Similar *Ri*_*g*_ patterns (Fig. 2e) implied that shear instability played an important role in driving upper layer dissipation. In contrast to *ε*, the *κ* (Fig. 2b) of all three layers was weakest in the upper layer (10^–6^–10^–5^ m^2^s^−1^), where *κ* was suppressed by strong stratification (Fig. 2c). The maximum *κ* in the upper layer of the LS (C13) reached 4.55 × 10^–5^ m^2^s^−1^, which is of the same order of magnitude as that at the other stations. Our results are different from the fine-scale parameterization results^18,19^ but are similar to the direct measurement results^16^. Turbulence and shear were relatively calm in the middle layer, with *ε* being mostly *O*(10^–10^) Wkg^−1^ and *ε* decreasing with depth. However, the *κ* in the middle layer (10^–5^ to 10^–4^ m^2^s^−1^) was generally larger than that in the upper layer and increased with depth due to weakening of stratification below the thermocline^24^.

A notable feature of the cross-section is that the bottom layer turbulence is significantly enhanced over rough topography across the LS (C13), with the maximum *ε* and *κ* values reaching 4.29 × 10^–8^ Wkg^−1^ and 3.7 × 10^–2^ m^2^s^−1^, respectively, which is comparable to previous work^25,26^. The *ε* and *κ* on the slope (C37) were also enhanced, with maximum values of 1.74 × 10^–8^ Wkg^−1^ and 3.5 × 10^–2^ m^2^s^−1^, respectively. Turbulence activated over rough topography is considered directly related to high-mode internal tides breaking close to topography and low-mode internal tides breaking over near-critical steep slopes^3^.

### Horizontal distributions of turbulence in different layers

**Figure 3:**
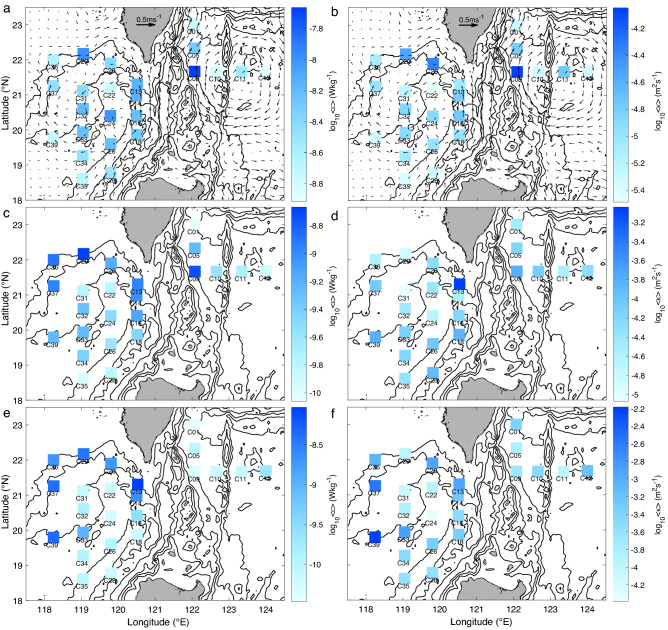
Maps of the layer-averaged dissipation rate (< *ε* > , left) and diapycnal diffusivity (< *κ* > , right) for the upper layer (**a**,**b**), middle layer (**c**,**d**), and bottom layer (**e**,**f**). Mean surface geostrophic currents from May 29 to June 24, 2018, are shown in (**a**) and (**b**). Figures were plotted using MATLAB R2016b (http://www.mathworks.com/).

The results presented above reveal that turbulence changes dramatically with depth, with *ε* ranging from 10^–10^ to 10^–7^ Wkg^−1^. Given the above fact, we choose to calculate the layer-averaged dissipation rate (< *ε* >) and diapycnal diffusivity (< *κ* >) to study the horizontal distributions of turbulence (Fig. 3). The averaged dissipation rate in the upper layer (< *ε*_upper_ >) of the WP was relatively low compared with those in the NSCS, except for the enhanced < *ε*_upper_ > at C09 and C05 (Fig. 3a). Mesoscale eddies and the Kuroshio are major phenomena in this area that may influence the intensified turbulence in the upper layer of the NSCS and WP^27,28^. In the upper layer near the thermocline, except for the geostrophic shear of mesoscale eddies and the Kuroshio, many other mechanisms or physical processes can drive turbulent mixing, such as near-inertial waves, internal tides, and subthermocline eddies^29,30^. However, the < *ε*_upper_ > values of the NSCS and WP were of the same order of magnitude. The pattern of < *κ* > in the upper layer (Fig. 3b) of the NSCS-WP area was similar to that of < *ε*_upper_ > because the distributions of *N*^2^ in the NSCS above the thermocline were comparable to those in the WP.

The average dissipation rates in the middle layer (< *ε*_middle_ >) of the NSCS and WP were one order of magnitude lower than those in the upper layer (Fig. 3c). In the WP, the pattern of < *ε*_middle_ > was similar to that in the upper layer, indicating that the influence of a large mesoscale eddy can reach deep depths to the middle layer, which can be seen in detail in Fig. 2a. The distribution also shows that < *ε*_middle_ > was enhanced at the NSCS slope and LS, but < *ε*_middle_ > in the NSCS basin was as weak as that in the WP, which was close to the background value. Because *N*^2^ decreases with depth, < *κ* > in the middle layer was not slope-enhanced considering that the slope was shallower than the basin (Fig. 3d).

The averaged dissipation rates in the bottom layer (< *ε*_bottom_ >) reveal clear horizontal patterns in the NSCS (Fig. 3e). The distribution of < *ε*_bottom_ > shows enhanced turbulence around the NSCS slope and the northern part of the LS, with a maximum *ε* at C13 reaching 10^–8^ Wkg^−1^, which was similar to that in the upper layer. A notable feature is that the < *ε*_bottom_ > in the northern part of the LS (C13) was two orders of magnitude higher than that in the southern part of the LS (C18), which was similar to the background values in the open oceans. This variability reveals that turbulence in the LS was nonuniform. The distribution of < *ε*_bottom_ > in the NSCS was also nonuniform, with a high < *ε*_bottom_ > in the slope area in the northern part and a low < *ε*_bottom_ > in the southern part, which is the NSCS basin. The < *ε*_bottom_ > in the NSCS basin was *O*(10^–10^) Wkg^−1^. The distribution of averaged < *κ* > in the bottom layer (Fig. 3f) followed the pattern of < *ε*_bottom_ > and was influenced by depth-correlated *N*^2^.

### Comparison of the turbulence activities of the NSCS and WP

**Figure 4:**
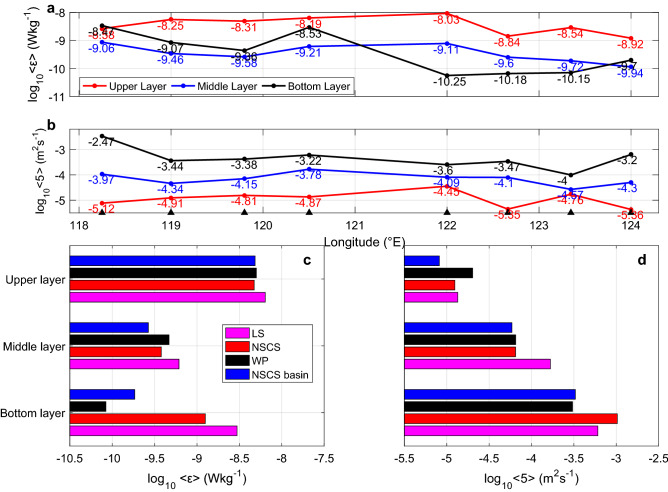
Zonal distribution of < *ε* > and < *κ* > (**a**,**b**) and comparison of averaged dissipation rates and diapycnal diffusivities for different areas (**c**,**d**) in each layer. The average values of the LS, WP and NSCS basin are calculated based on stations in the same color in Fig. 1. The NSCS includes stations of the NSCS slope and NSCS basin in Fig. 1.

To clarify the difference between the turbulence activities of the NSCS and WP, we first present the zonal distributions of < *ε* > (Fig. 4a) and < *κ* > (Fig. 4b) in the NSCS and WP. In the zonal direction, the variations in < *ε*_upper_ > and < *ε*_middle_ > have similar trends, with values of 10^–8^ to 10^–9^ Wkg^−1^ and 10^–9^ to 10^–10^ Wkg^−1^, respectively. A slight increase was observed near 122°E, which was considered the effect of eddy-induced turbulence. < *ε*_bottom_ > shows a different pattern from < *ε*_upper_ > , with significant increases at the LS (122.5° E) and 118.25° E, indicating the effects of topography. The zonal distribution of < *κ* > was similar to that of < *ε* > . < *κ* > was not as high as that reported by previous indirect measurements^18,19^, which may be induced by uncertainties of the fine-scale method near rough topography^31^.

The average dissipation rates and diapycnal diffusivity of different layers are shown in Fig. 4c,d to evaluate the differences between the WP (< *ε*_WP_ > and < *κ*_WP_ >), LS (< *ε*_LS_ > and < *κ*_LS_ >), NSCS basin (< *ε*_NSCSbasin_ > and < *κ*_NSCSbasin_ >) and NSCS (< ε_NSCS_ > and < κ_NSCS_ >) considering the horizontal pattern shown in Fig. 3. Subscripts LS, WP and NSCS correspond to stations in the same color in Fig. 1. The NSCS subscript represents both the NSCS slope and NSCS basin in Fig. 1. Turbulence was more energetic in the LS than in the NSCS and WP at all layers, as shown in previous work^19^. The ratios of < *ε*_NSCS_ > to < *ε*_WP_ > in the upper, middle and bottom layers were 0.94, 0.81 and 14.89, respectively, indicating that the dissipation rates were at the same level in the upper and middle layers, while in the bottom layer, < *ε*_NSCS_ > was one order of magnitude larger than < *ε*_WP_ > . The ratios of < *κ*_NSCS_ > to < *κ*_WP_ > in the upper, middle and bottom layers were 0.62, 0.99, and 3.38, respectively, showing that the diffusivities in the NSCS were smaller than or equal to those in the WP in the upper and middle layers. Enhanced diffusivities were observed in the bottom layer of the NSCS and were larger than those in the WP by a factor of 3 instead of by 2 orders of magnitude^18^. The ratios of < *ε*_NSCSbasin_ > to < *ε*_WP_ > in the upper, middle and bottom layers were 0.96, 0.57 and 2.06, respectively, revealing the same level of *ε* in the NSCS basin and WP. The ratios of < *κ*_NSCSbasin_ > to < *κ*_WP_ > in the upper, middle and bottom layers were 0.40, 0.90, and 1.08, respectively. Surprisingly, both the dissipation rates and diffusivities of all three layers of the NSCS basin and WP were similar.

Considering the influence of the definition of different layers, we also calculated the average ε and κ at different layers based on depth (see Supplementary Figs. S1 and S2 online). The distributions of ε at the upper (50–500 m) and bottom layers (within 500 MAB), defined based on depth, are similar to those in Figs. 3 and 4. A dramatic difference in κ is shown in the middle layer (500–1500 m) because the depth in the NSCS is shallower than that in the WP. This results in a comparison of bottom-enhanced turbulence in the NSCS with mild turbulence in the middle layer of the WP, and this comparison is unfair.

## Discussion

### Temporal variation in dissipation rates

Given that the occurrence of turbulence activity is intermittent and typically has strong temporal variations due to different forcing mechanisms^30^, it is quite challenging to give a robust horizontal distribution of turbulent mixing based on one instantaneous observation or observations at different times. Compared to instantaneous microstructure measurements, the moored ADCP is more effective in continuously observing oceanic fine-scale velocity profiles at a fixed location and hence estimating fine-scale turbulent shear and its resultant mixing^32,33^. To clarify the confidence of our study, the GHP method was applied to ADCP data to estimate the time series of ε and to evaluate the variation in ε.

**Figure 5:**
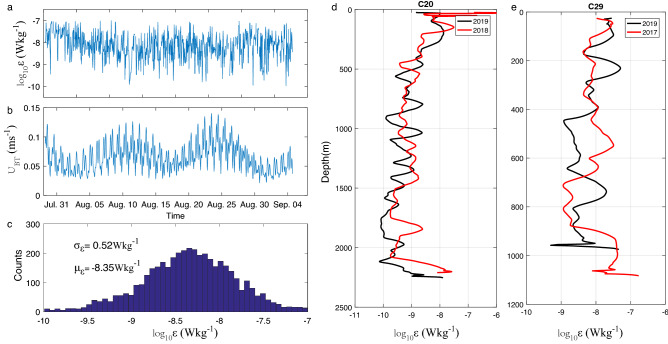
Panels (**a**,**b**) are the time series of the dissipation rates and the synchronous barotropic tidal current. Panel (**c**) shows the histogram of the fine-scale dissipation rate during the moored ADCP observation period. μ_ε_ and σ_ε_ are the mean value and standard deviation of the normal distribution of dissipation rates in log10 space. Panels (**c**,**d**) are repeated microstructure observations at C20 and C29 in different years.

The results shown in Fig. 5a,b are examples of the bottom ε time series from July 31 to September 4, 2017, and the synchronous barotropic tidal current derived from the TPXO 7.2 inverse model (http://volkov.oce.orst.edu/tides/global.html). Dissipation rates show significant variations but are not highly correlated with tidal currents. The mean value (log_10_ μ_ε_) and standard deviation (log_10_ σ_ε_) of the normal distribution of dissipation rates are − 8.35 Wkg^−1^ and 0.52 Wkg^−1^, respectively (Fig. 5c). The mean value is very close to the directly observed bottom dissipation rate at C29 in log10 space, which is − 8.42 Wkg^−1^. The standard deviation also indicates that most values could distribute within one order of magnitude of the mean value. The standard deviation can support the study of dissipation rate distribution with orders of magnitude variation.

We also took repeated VMP-X casts in different years at C29 and C20. Repeat casts at C20 were taken on June 06, 2018, and July 27, 2019. The results showed that the values and vertical structures of ε (Fig. 5d) are similar. Both profiles present enhanced ε in the upper and bottom layers and low ε in the middle layer. Repeat casts at C29 (Fig. 5e) were taken on December 10, 2017, and July 21, 2019. The depth changed 107 m between the two deployments because of complicated bathymetry near the LS, although the two casts were very close to each other. The structures of the two profiles were also similar to each other, with bottom-enhanced dissipation rates and ε values on the same order of magnitude. Thus, both the time series of bottom ε from the fine-scale method and the repeat microstructure measurements indicate that the distribution of turbulence activities based on our direct measurements is credible.

### Effect of topography on the dissipation rate distribution

**Figure 6:**
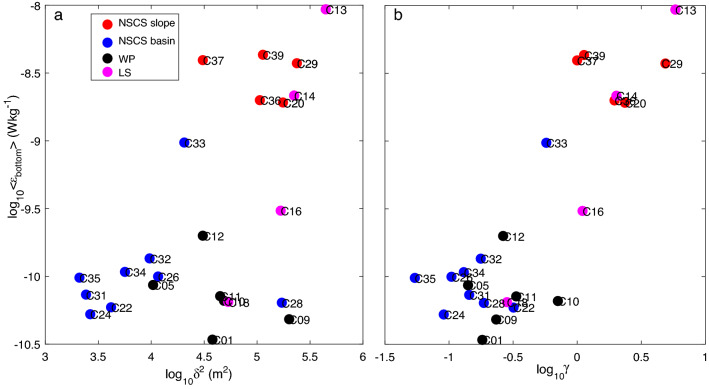
Average dissipation rate at the bottom layer (< *ε*_bottom_ >) plotted against topography roughness (*δ*^2^) and slope criticality (*γ*) for semidiurnal tides. These symbols correspond to the station symbols in Fig. 1.

Turbulence in the SCS is revealed to be remarkably enhanced, mainly due to internal tides, which are generated from the LS and propagate westward into the SCS^9,34^. High-mode internal tides dissipate locally in the near-field, and low-mode internal tides propagate to the far-field^35^. As internal waves propagate, variable bathymetry plays an important role in the transfer of low-mode energy to smaller scales, at which dissipation occurs^36^. Since dissipation rates were enhanced mainly in the bottom layer, we present the relationship between < *ε*_bottom_ > and topography parameters such as topography roughness (*δ*^2^, Fig. 6a) and slope criticality for semidiurnal tides (*γ*, Fig. 6b). This enhancement, however, does not occur throughout the entire NSCS but mainly in sloped and rough areas. The topography of the NSCS basin is relatively smooth, with a low < *ε* > of *O*(10^–10^) Wkg^−1^. A high < *ε* > of *O*(10^–8^) Wkg^-1^ was combined with rough topography at the NSCS slope and LS. The high roughness corresponded to high-mode internal tides dissipating locally^22^. Several stations in the WP and LS with higher roughness showed low dissipation rates because most internal tide energy is not dissipated near topographic sources and instead radiates away as low-mode internal waves^3^. In the NSCS basin, internal tides propagated in a low mode with less interaction with smooth topography, showing low dissipation^9^. The interaction between low-mode internal waves and rough topography is strongly dependent on the steepness of the topography^37^. When internal tides shoal onto the continental slope, near-critical topography (*γ* ~ 1) scatters low-mode internal tides to smaller wavelengths, leading to intensified dissipation near the slope^38,39^. Thus, < *ε*_bottom_ > (Fig. 6b) was highly related to slope criticality for semidiurnal tides, showing high dissipation near the slope and low dissipation in the basin.

We present a clear picture of the three-dimensional distribution of turbulence based on full-depth microstructure data taken at the NSCS and WP. Our analyses show that in the upper and middle layers, turbulence activities in the NSCS and WP were of the same order of magnitude, with < *ε* > ranging from 10^–8^ to 10^–9^ Wkg^−1^ and < *κ* > ranging from 10^–5^ to 10^–6^ m^2^s^−1^. Enhanced turbulence was observed only near rough topography and steep slopes in the bottom layer, with the average dissipation rates of the NSCS being one order of magnitude larger than those of the WP. However, the enhanced diffusivities in the bottom layer of the NSCS were larger than those in the bottom layer of the WP by a factor of 3 instead of by 2 orders of magnitude. This enhancement, however, does not occur throughout the entire NSCS but mainly at the slope and little in the basin. The distribution of turbulence in the bottom layer was mainly induced by the breaking of internal tides at the slope and propagation in the basin. To our knowledge, this study was the first to compare the turbulence activities of the NSCS and WP based on direct full-depth microstructure observations. These results may appear surprising in light of previous studies but are in fact consistent with predictions from internal wave-topography interaction theory. Since the topography near LS covers a wide range of bathymetric features, we speculate that the relationship between direct observed dissipation and topography may be applied to the global ocean.

## Methods

### Microstructure estimates of turbulent dissipation

The microstructure data were analyzed by following previous work by Wolk et al. (2002)^40^. The dissipation rate was estimated by fitting the Nasmyth spectrum to the measured shear spectra over consecutive half-overlapping 10-m depth segments; thus, the vertical resolution of *ε* was 5 m. The same spacing was adopted for profiles of the potential temperature and shear. Diapycnal diffusivity (*κ*) was estimated by following the Osborn (1980)^41^ formula *κ* = *Γε*/*N*^2^, where *Γ* is the mixing efficiency, and *Γ* = 0.2 is typically adopted. Mixing efficiency may vary significantly in the abyssal ocean^42,43^, while there is no widely accepted value of *Г* and method besides *Г* = 0.2. We choose *Г* = 0.2 in this study because this study focuses on the distribution of the dissipation rate rather than diapycnal diffusivity, which is highly dependent on depth. Buoyancy frequency was calculated as $N = \sqrt { - \frac{g}{{\rho_{0} }}\frac{d\rho }{{dz}}}$. The estimated *ε* and *κ* values in the top 10 m were removed due to contamination by the ship. Velocity shear $S = \sqrt {({{\partial u} \mathord{\left/ {\vphantom {{\partial u} {\partial z}}} \right. \kern-\nulldelimiterspace} {\partial z}})^{2} + ({{\partial v} \mathord{\left/ {\vphantom {{\partial v} {\partial z}}} \right. \kern-\nulldelimiterspace} {\partial z}})^{2} }$, where *u* and *v* represent the zonal and meridional components from the LADCP, respectively. *N*^2^ and S^2^ were interpolated to a 10-m vertical spacing, corresponding to the resolution of *ε*. The gradient Richardson number, $Ri_{g} = \frac{\partial \rho }{{\partial z}}\frac{g}{{\rho_{o} }}\left( {\frac{{\partial \overline{u}}}{\partial z}} \right)^{ - 2}$, is the ratio of the stabilizing forces of density stratification to the destabilizing influences of velocity shear.

### GHP parameterization

GHP parameterization^44^ depends on the fine-scale shear $\left\langle {v_{z}^{2} } \right\rangle$ and strain variance $\left\langle {\xi_{z}^{2} } \right\rangle$ as.

1 $$\varepsilon_{{{\text{GHP}}}} { = }\varepsilon_{0} (\frac{{N^{2} }}{{N_{0}^{2} }})\frac{{\left\langle {v_{z}^{2} } \right\rangle^{2} }}{{_{GM} \left\langle {v_{z}^{2} } \right\rangle^{2} }}h(R_{w} )j(\frac{f}{{\overline{N} }})$$

2 $$h(R_{w} ) = \frac{3}{2\sqrt 2 }\frac{{R_{w} + 1}}{{R_{w} \sqrt {R_{w} - 1} }}$$

3 $$j(\frac{f}{{\overline{N} }}){ = }\frac{f\arccos h(N/f)}{{f_{30} \arccos h(N_{0} /f_{30} )}}$$

4 $$R_{w} { = }\left\langle {v_{z}^{2} } \right\rangle /\overline{N}^{2} \left\langle {\xi_{z}^{2} } \right\rangle$$

where *ε*_0_ = 8 × 10^–10^ Wkg^−1^ is a background constant value; *N* is the buoyancy frequency, with *N*_0_ = 5.42 × 10^–3^ rads^−1^; *f* is the Coriolis frequency, with *f*_30_ = *f*(30°); $\left\langle {v_{z}^{2} } \right\rangle$ is the shear variance; $_{GM} \left\langle {v_{z}^{2} } \right\rangle$ is the shear variance from the GM model spectrum; and *R*_w_ is the ratio of the buoyancy-frequency-normalized shear variance to strain variance. Here, *R*_w_ is set to 7^45^. The strain variance $\left\langle {\xi_{z}^{2} } \right\rangle$ is estimated from the buoyancy frequency, $\left\langle {\xi_{z}^{2} } \right\rangle { = }\left\langle {{{\left( {N^{2} - \overline{N}^{2} } \right)^{2} } \mathord{\left/ {\vphantom {{\left( {N^{2} - \overline{N}^{2} } \right)^{2} } {\overline{N}^{4} }}} \right. \kern-\nulldelimiterspace} {\overline{N}^{4} }}} \right\rangle$.

To compute the buoyancy-frequency-normalized shear variance,$\left\langle {v_{z}^{2} } \right\rangle /\overline{N}^{2}$, the shear profiles were first obtained from the hourly 75 kHz ADCP velocity data with 8 m depth resolution and the buoyancy frequency profiler from the global physical analysis and a coupled forecasting product^46^ modeled by the Met Office Coupled Atmosphere–Land–Ocean–Ice data assimilation system (CPLDA). The vertical shear spectrum of the velocity in the segment from 848 to 1196 m was calculated. Then, the buoyancy frequency-normalized shear variance, $\left\langle {v_{z}^{2} } \right\rangle /N^{2}$, was obtained by integrating the shear spectrum from the minimum wavenumber *k*_min_ = 2π/180 m to *k*_max_ = 2π/60 m^47,48^. $_{GM} \left\langle {v_{z}^{2} } \right\rangle$ was also obtained within the same integral interval.

6 $$\frac{{\left\langle {v_{z}^{2} } \right\rangle }}{{\overline{N}^{2} }} = \int_{{k_{\min } }}^{{k_{\max } }} {S[\frac{{v_{z} }}{{\overline{N} }}} ](k_{z} )dk_{z}$$

### Characteristics of topography

Bathymetry data from the General Bathymetric Chart of the Oceans 2014 were used to evaluate topography roughness *δ*^2^ (Fig. 1b) and slope criticality *γ* for semidiurnal tides (Fig. 1c). Topography roughness is defined as the variance calculated in a 0.2° × 0.2° area, a reasonable scale for internal tide generation^49^. Slope criticality is defined as *γ* = (*dh*/*dx*)/*s*, where *dh*/*dx* is the topographic gradient and $s = \sqrt {(f^{2} - \omega^{2} ) - (N^{2} - \omega^{2} )}$ is the internal tide characteristic steepness, where *ω* is the internal wave frequency and *f* is the Coriolis parameter^39^. The climatological temperature and salinity data in the 2013 World Ocean Atlas were used to calculate the *N*^2^ used in *γ* (Fig. 1c).

## Supplementary Information

Supplementary Information.

## Data Availability

Bathymetry data from the General Bathymetric Chart of the Oceans 2014 can be downloaded from the website https://www.gebco.net/data_and_products/historical_data_sets/#gebco_2014. The climatological temperature and salinity data in the 2013 World Ocean Atlas are obtained from https://www.nodc.noaa.gov/OC5/woa13/woa13data.html. The geostrophic currents are obtained from http://marine.copernicus.eu/. Direct measurements of microstructure data, squared buoyancy frequency and shear variance are available from the corresponding author upon request. The buoyancy frequency data are calculated from the global physical analysis and a coupled forecasting product by the Met Office Coupled Atmosphere–Land–Ocean–Ice data assimilation system, which can be obtained from https://resources.marine.copernicus.eu/?option=com_csw&view=details&product_id=GLOBAL_ANALYSISFORECAST_PHY_CPL_001_015.
