# Supplementary material for: Observed three dimensional distributions of enhanced turbulence near the Luzon Strait
Source: Sci Rep. 2021 Jul 21;11:14835. doi: 10.1038/s41598-021-94223-3 (PMC8295335; doi:10.1038/s41598-021-94223-3)
Supplement: Supplementary file 1 — Supplementary Information. [file 41598_2021_94223_MOESM1_ESM.docx]

Scientific Reports
Supplementary Information for

**Observed three dimensional distributions of enhanced turbulence near the Luzon Strait**

Jianfeng Wang^1, 2, 3^, Fei Yu^1, 2, 3, 4*^, Feng Nan^1, 2, 3^, Qiang Ren^1, 2^, Zifei Chen^1, 4^, and Tongtong Zheng^1, 4^

^1^CAS Key Laboratory of Ocean Circulation and Waves, Institute of Oceanology, Chinese Academy of Sciences, Qingdao 266071, China;

^2^ Center for Ocean Mega-Science, Chinese Academy of Sciences, Qingdao 266071, China;

^3^ Pilot National Laboratory for Marine Science and Technology (Qingdao), Qingdao 266237, China;

^4^ University of Chinese Academy of Sciences, Beijing 100049, China.

**(Submitted to Scientific Reports)**

^*^Corresponding author:

Dr. Fei Yu
Institute of Oceanology, Chinese Academy of Sciences

Nanhai Road, Qingdao 266071, China

Phone: (86)053282898187; Fax: (86)053282898186

E-mail: [*yuf@qdio.ac.cn*](mailto:yuf@qdio.ac.cn)

**Supplementary Information included in this file:**

Supplementary Figures S1 and S2

Considering the influence of definition of different layers, we also calculated the average ε and κ following Yang et al. (2016) which is based on depth (Fig. S1 and S2). Distributions of ε at upper and bottom layers, defined based on depth, are similar to that in Fig. 3 and 4. Dramatic difference of κ showed in the middle layer, because depth in NSCS is shallower than that in WP. This results in comparison of bottom enhanced turbulence in NSCS with mild turbulence in the middle layer WP and this comparison is unfair.


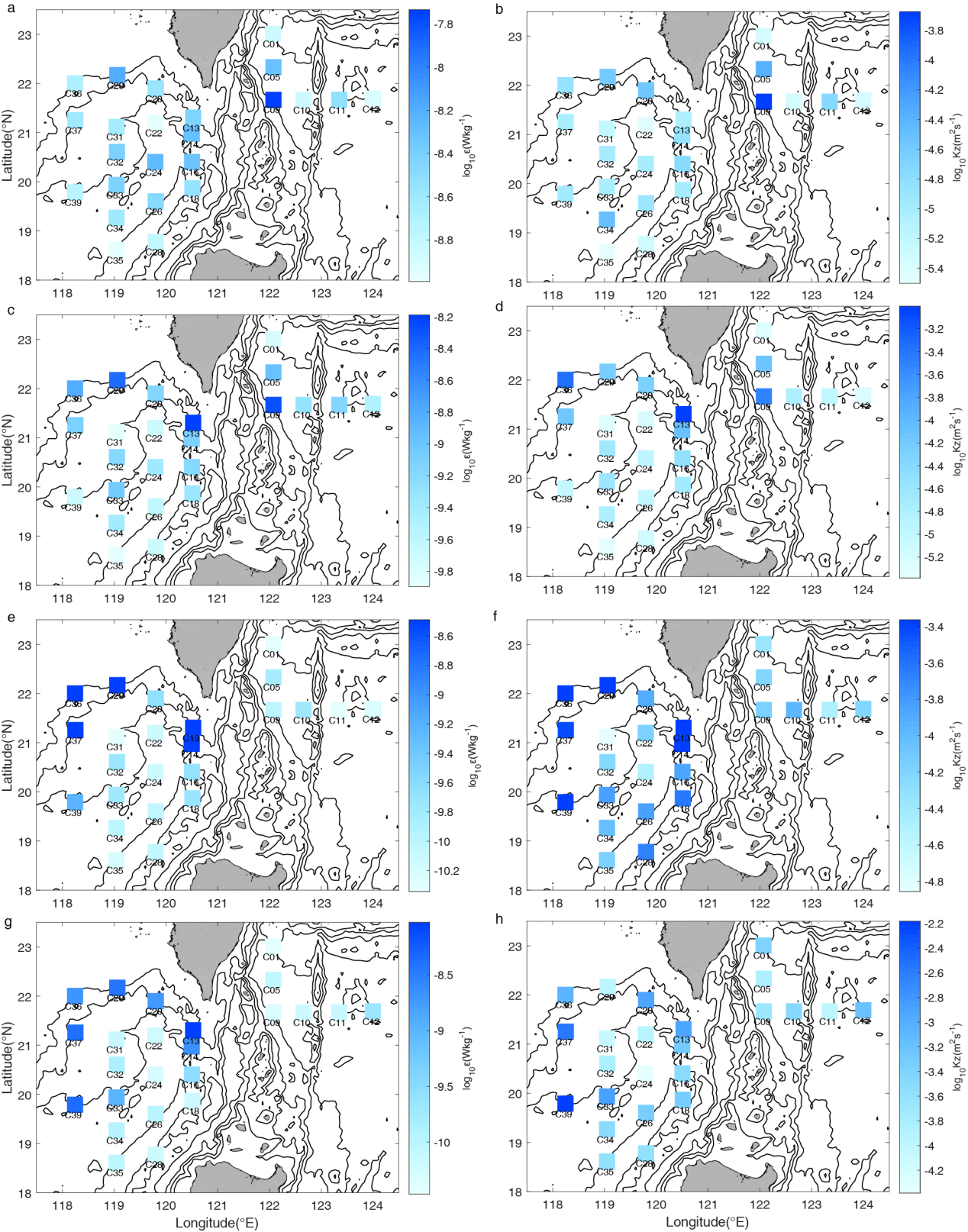


**Figure S1.** Maps of the layer-averaged dissipation rate (<*ε*>, left) and diapycnal diffusivity (<*κ*>, right) for the upper layer (a and b, 50-500m), middle layer (c and d 500-1500m), deep layer (e and f, 1500-500 MAB) and bottom layer (g and h, within 500 MAB).


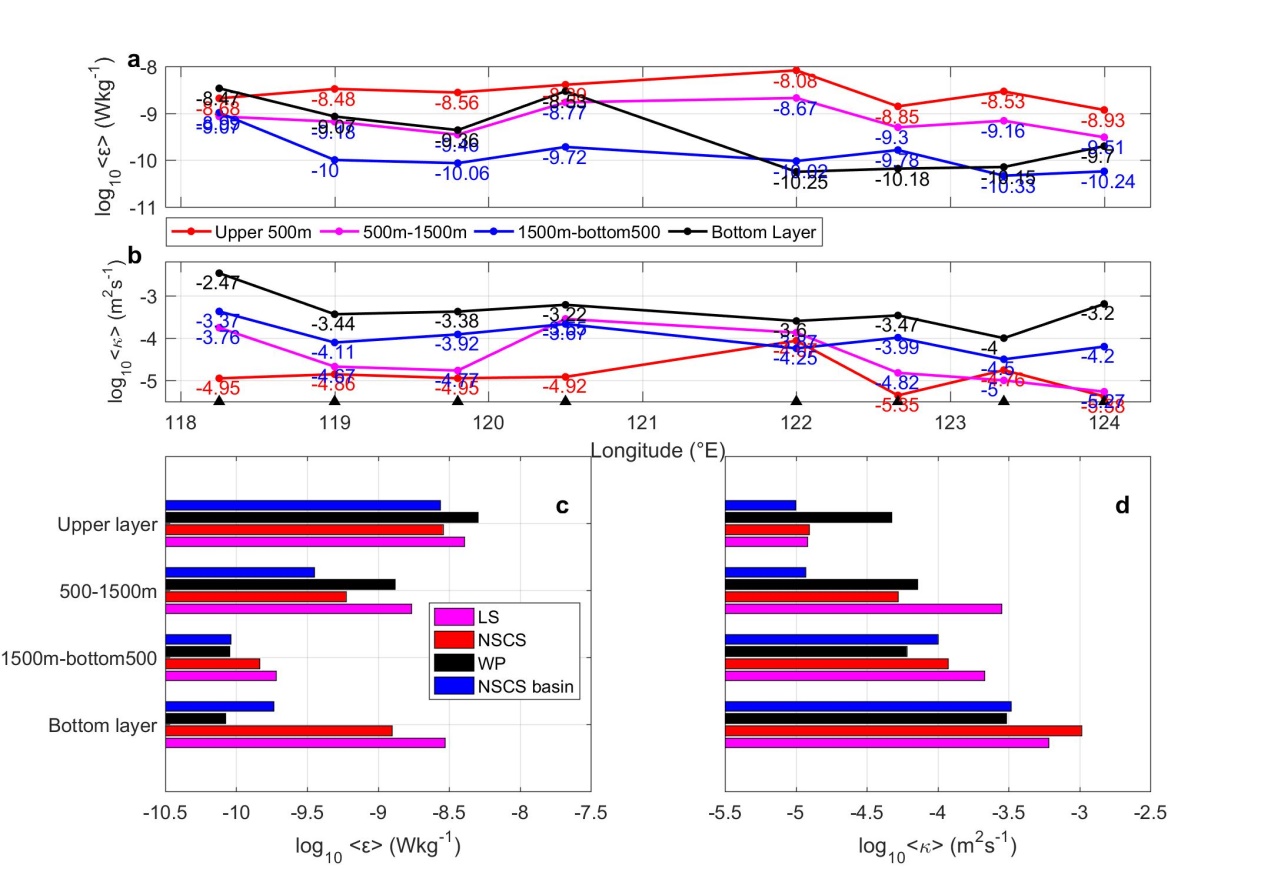


**Figure S2.** Zonal distribution of <*ε*> and <*κ*> (a and b), and comparison of averaged dissipation rate and diapycnal diffusivity for different areas (c and d) in each layer. Average value of LS, WP and NSCS basin are calculated on the basis of stations in the same color in Figure 1. NSCS includes stations of NSCS slope and NSCS basin in Figure 1.
